# Análise de *fake news* veiculadas durante a pandemia de COVID-19 no Brasil

**DOI:** 10.26633/RPSP.2021.65

**Published:** 2021-05-13

**Authors:** Thainá do Nascimento de Barcelos, Luíza Nepomuceno Muniz, Deborah Marinho Dantas, Dorival Fagundes Cotrim, João Roberto Cavalcante, Eduardo Faerstein

**Affiliations:** 1 Universidade do Estado do Rio de Janeiro (UERJ), Instituto de Nutrição Rio de Janeiro (RJ) Brasil Universidade do Estado do Rio de Janeiro (UERJ), Instituto de Nutrição, Rio de Janeiro (RJ), Brasil.; 2 Universidade do Estado do Rio de Janeiro (UERJ), Instituto de Ciências Sociais Rio de Janeiro (RJ) Brasil Universidade do Estado do Rio de Janeiro (UERJ), Instituto de Ciências Sociais, Rio de Janeiro (RJ), Brasil.; 3 Universidade do Estado do Rio de Janeiro (UERJ), Instituto de Medicina Social Rio de Janeiro (RJ) Brasil Universidade do Estado do Rio de Janeiro (UERJ), Instituto de Medicina Social, Rio de Janeiro (RJ), Brasil.

**Keywords:** Infecções por Coronavírus, acesso à Internet, meios de comunicação, mídias sociais, saúde global, saúde pública, Brasil, Coronavirus infections, internet access, communications media, social media, global health, public health Brazil, Infecciones por Coronavirus, acesso a internet, medios de comunicación, medios de comunicación sociales, salud global, salud pública, Brasil

## Abstract

**Objetivo.:**

Caracterizar as *fake news* sobre COVID-19 que circularam no Brasil de janeiro a junho de 2020.

**Métodos.:**

As *fake news* registradas até 30 de junho de 2020 em dois *sites* (G1, da corporação Globo, e Ministério da Saúde) foram coletadas e categorizadas de acordo com o seu conteúdo. Para cada notícia enganosa, foram extraídos os seguintes dados: data de circulação, título, canal de divulgação (por exemplo, WhatsApp), formato da divulgação (por exemplo, texto, foto ou vídeo) e portal de registro. Os termos encontrados nos títulos das notícias falsas foram analisados no Google Trends para determinar se houve aumento de buscas no Google com utilização desses termos após a disseminação de uma determinada notícia enganosa. Foram também identificadas as macrorregiões brasileiras com maior porcentagem de aumento nas buscas utilizando os termos analisados.

**Resultados.:**

Foram identificadas 329 *fake news* relacionadas à pandemia de COVID-19 nos *sites* estudados (253 no G1 e 76 no Ministério da Saúde). As *fake news* foram disseminadas principalmente através de WhatsApp e Facebook. As categorias temáticas mais frequentes foram: política (por exemplo, governantes falsificando a vacinação contra a COVID-19, com 20,1%), epidemiologia e estatística (proporção dos casos e óbitos, 19,5%) e prevenção (16,1%). Conforme o Google Trends, houve um aumento de 34,3% nas buscas que utilizavam termos presentes nas *fake news.* O maior aumento nas buscas ocorreu no Sudeste (45,1%) e Nordeste (27,8%).

**Conclusões.:**

As *fake news* divulgadas durante os primeiros 6 meses da pandemia de COVID-19 no Brasil se caracterizaram por conteúdos de posicionamento político e desinformação sobre número de casos e óbitos e medidas de prevenção e de tratamento. Os principais veículos de divulgação foram o WhatsApp e o Facebook, com utilização de mensagens, imagens e vídeos, tendo maior alcance nas regiões Sudeste e Nordeste do país.

## Abstract (en)

**Objective.:**

To describe the characteristics of fake news about COVID-19 disseminated in Brazil from January to June 2020.

**Method.:**

The fake news recorded until 30 June 2020 in two websites (Globo Corporation website G1 and Ministry of Health) were collected and categorized according to their content. From each piece of fake news, the following information was extracted: publication date, title, channel (e.g., WhatsApp), format (text, photo, video), and website in which it was recorded. Terms were selected from fake news titles for analysis in Google Trends to determine whether the number of searches using the selected terms had increased after the fake news appeared. The Brazilian regions with the highest percent increase in searches using the terms were also identified.

**Results.:**

In the two websites, 329 fake news about COVID-19 were retrieved. Most fake news were spread through WhatsApp and Facebook. The most frequent thematic categories were: politics (20.1%), epidemiology and statistics (e.g., proportion of cases and deaths) (19.5%), and prevention (16.1%). According to Google Trends, the number of searches using the terms retrieved from the fake news increased 34.3% during the period studied. The largest increase was recorded in the Southeast (45.1%) and the Northeast (27.8%).

**Conclusions.:**

The fake news spread during the first 6 months of the COVID-19 pandemic in Brazil were characterized by political content as well as misinformation about the number of cases and deaths and about prevention measures and treatment. The main dissemination channels were WhatsApp and Facebook, with the use of messages, images, and videos, with greater reach in the Southeast and Northeast of Brazil.

## RESUMEN

**Objetivo.:**

Analizar algunas características de las noticias falsas sobre la COVID-19 en circulación en Brasil de enero a junio del 2020.

**Métodos.:**

Se recolectaron las noticias falsas registradas hasta el 30 de junio del 2020 en dos sitios (G1, perteneciente a la Corporación Globo, y el Ministerio de Salud) y se clasificaron de acuerdo con su contenido. Se extrajeron los siguientes datos de cada noticia engañosa: fecha de circulación, título, canal (por ejemplo, WhatsApp) y formato de divulgación (texto, fotografía o video) y portal de registro. Se analizaron en Google Trends los términos encontrados en los títulos de las noticias falsas para determinar si había aumentado el número de búsquedas en Google con esos términos después de la difusión de una noticia engañosa. También se determinaron las macrorregiones brasileñas con el mayor aumento porcentual en las búsquedas hechas con los términos analizados.

**Resultados.:**

Se encontraron 329 noticias falsas relacionadas con la pandemia de COVID-19 en los sitios estudiados (253 en el G1 y 76 en el Ministerio de Salud). Esas noticias se divulgaron principalmente por medio de WhatsApp y Facebook. Las categorías temáticas más frecuentes fueron política (por ejemplo, falsificación de la vacuna contra la COVID-19 por los gobernantes, 20,1%), epidemiología y estadística (proporción de casos y muertes, 19,5%) y prevención (16,1%). Según Google Trends, aumentaron un 34,3% las búsquedas en las cuales se utilizaban términos contenidos en las noticias falsas. El mayor aumento de esa clase se registró en el sudeste (45,1%) y el nordeste (27,8%) del país.

**Conclusiones.:**

Las noticias falsas divulgadas durante los seis primeros meses de la pandemia de COVID-19 en Brasil se caracterizaron por contenidos de posicionamiento político y desinformación sobre el número de casos y muertes y sobre las medidas de prevención y tratamiento. Los principales medios de divulgación fueron WhatsApp y Facebook, con utilización de mensajes, imágenes y videos, y un mayor alcance en las regiones sudeste y nordeste del país.

Após a notificação de casos de pneumonia de causa desconhecida em 31 de dezembro de 2019 na cidade chinesa de Wuhan, identificou-se a circulação de uma nova variante do coronavírus (SARS-CoV-2), posteriormente associada à doença denominada COVID-19 (1, 2). Tendo em vista a rápida expansão da COVID-19 a outros países, a Organização Mundial da Saúde (OMS) declarou Emergência de Saúde Pública de Importância Internacional em 30 de janeiro de 2020 e, em 11 de março, reconheceu a existência de pandemia (3, 4).

**FIGURA 1.**
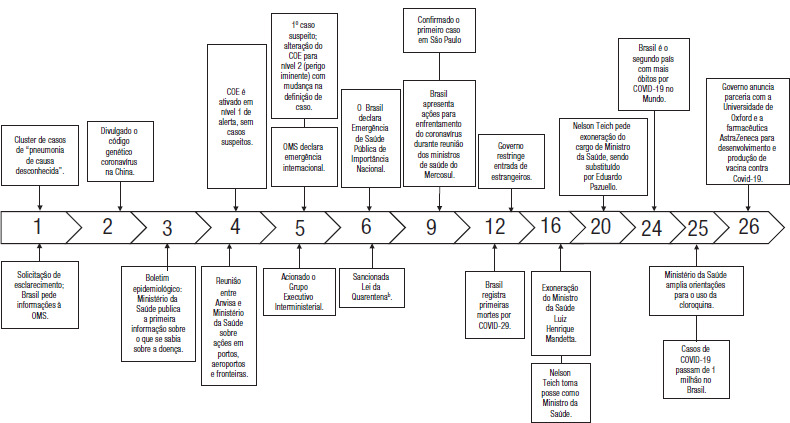
Linha do tempo de acontecimentos sobre a pandemia de COVID-19 no Brasil nas semanas epidemiológicas de 1 a 26, 2020^a^

***Fonte:*** G1 — Fato ou Fake — Coronavírus; e Ministério da Saúde — Fake News — Coronavírus.

a Anvisa: Agência Nacional de Vigilância Sanitária; COE: Comitê de Operações de Emergência; OMS: Organização Mundial da Saúde. Primeira semana epidemiológica: 4 de janeiro de 2020; última semana epidemiológica: 27 de junho de 2020.

^b^Lei da Quarentena: lei no. 13 979/2020, sancionada em fevereiro de 2020 para dispor sobre “medidas para enfrentamento da emergência de saúde pública de importância internacional decorrente do coronavírus responsável pelo surto de 2019”. Nela estão previstos os mecanismos que podem ser utilizados pelas autoridades sanitárias a fim de conter o avanço da pandemia, entre eles a quarentena e o isolamento.

No Brasil, os primeiros casos suspeitos de COVID-19 foram notificados entre 18 e 27 de janeiro de 2020. Como mostra a figura 1, em 22 de janeiro, foi ativada a estratégia prevista no Plano Nacional de Resposta às Emergências em Saúde Pública (5) e, em 3 de fevereiro, foi declarada Emergência em Saúde Pública de Importância Nacional pelo governo brasileiro (6). Até 26 de fevereiro de 2021, haviam sido confirmados no país 10 393 886 casos e 251 661 óbitos (7).

A gestão da grave situação sanitária em que se transformou a COVID-19 foi, entretanto, complicada por outro cenário incidente: a rápida disseminação global de informações (8). Na atual era digital, a drástica diminuição dos custos e esforços necessários à geração e à divulgação de informações e opiniões possibilita, de um lado, o acesso do público em geral a mídias digitais e sociais confiáveis como fonte de informação (por exemplo, páginas oficiais da OMS, do governo federal e de governos estaduais, de entidades de saúde e de órgãos de comunicação de massa); de outro, porém, permite também uma intensa propagação de notícias falsas — as chamadas *fake news* (9-11).

Embora não haja uma definição definitiva de *fake news*, com diversos autores ainda refletindo sobre o assunto, um conceito possível é o de “histórias falsas que parecem ser notícias, espalhadas na Internet ou usando outros meios de comunicação, geralmente criadas para influenciar visões políticas ou como uma piada” (12). Informações falsas disseminadas nas redes digitais e sociais são especialmente preocupantes para a saúde pública, visto que podem prejudicar a eficácia de programas, campanhas e iniciativas que visam à saúde e ao bem-estar dos cidadãos (13). No contexto da pandemia de COVID-19, um exemplo de *fake news* foram as crescentes campanhas antivacinas (14).

Segundo o mais recente Relatório de segurança digital no Brasil (15), em 2018 o país já estava entre aqueles com maior produção e circulação de *fake news* no mundo. Entre o primeiro e o segundo trimestres de 2018, houve um aumento de cerca de 50% na identificação dessas notícias (15). A pandemia de COVID-19 vem exacerbando esse fenômeno (16), o qual se tornou motivo de grande preocupação, especialmente diante do aumento progressivo de buscas na Internet sobre temas de saúde por parte da população, sendo o Google a ferramenta mais utilizada (17). A pesquisa referenciada da Avaaz (18) aponta que nove em cada 10 brasileiros entrevistados leram ou ouviram ao menos uma informação falsa sobre a COVID-19 e que sete em cada 10 acreditam em ao menos uma desinformação veiculada. Ainda, outra pesquisa apontou que 62% dos brasileiros não sabem reconhecer se uma mensagem é falsa ou verdadeira (19, 20).

Considerando que desconhecer, estar desinformado ou agir de má-fé podem levar indivíduos a prejudicarem os esforços de profissionais de saúde e de autoridades sanitárias engajadas no controle da pandemia (19, 21), o objetivo do presente artigo foi caracterizar as *fake news* sobre COVID-19 que circularam no Brasil de janeiro a junho de 2020.

## MATERIAIS E MÉTODOS

O presente estudo utilizou uma metodologia de revisão documental. Realizou-se um levantamento das *fake news* disponibilizadas em dois *sites*, G1 e Ministério da Saúde, de 1º de janeiro a 30 de junho de 2020. O portal de notícias G1 e o Ministério da Saúde criaram espaços exclusivos para receber informações sobre *fake news* e disponibilizaram números de telefone para uso da população.

As informações recebidas pelos portais através desses canais são investigadas e classificadas de acordo com sua veracidade nas seções “Fato ou Fake — Coronavírus” (https://g1.globo.com/fato-ou-fake/coronavirus/), no portal G1, e “Fake News” (https://antigo.saude.gov.br/component/tags/tag/novo-coronavirus-fake-news), no *site* do Ministério da Saúde.

A partir da análise dos dois portais, foram selecionadas as *fake news* relevantes, com extração e armazenamento em planilhas dos seguintes dados: data de circulação, título, canal de divulgação, formato de divulgação e portal (G1 ou Ministério da Saúde). Após a coleta, foram excluídas as duplicatas de *fake news* identificadas nos dois repositórios, sendo mantidas as inserções mais antigas.

De acordo com o seu conteúdo, as notícias foram agrupadas em categorias temáticas. A categorização do conteúdo das notícias foi feita por dois autores do artigo (TNB, LNM), levando em consideração o conteúdo apresentado pelas informações falsas. O resultado foi posteriormente comparado e, quando não houve concordância entre eles ou a categorização se apresentava de maneira dúbia, um terceiro autor foi consultado (DMD). Todos os autores aprovaram as categorias temáticas.

A ferramenta Google Trends também foi utilizada. Essa ferramenta permite o acompanhamento da evolução do número de buscas por uma determinada palavra-chave no Google e em *sites* relacionados, como o YouTube (22). Quando há dados suficientes, a ferramenta permite a geração de gráficos que mostram a frequência das buscas que incluem um determinado termo nas várias regiões do mundo e em vários idiomas (22). Além disso, é possível refinar a pesquisa segundo região geográfica, intervalo de tempo, categoria (ciência, saúde, esportes) e local de busca (Google shopping, Google notícias, Google imagens).

O Google Trends permite comparar até cinco grupos de termos simultaneamente para explorar o interesse *on-line* em cada um deles (22). Os termos “coronavírus” e “COVID-19” foram comparados para identificar qual entre os dois havia sido mais utilizado pelos usuários do Google no Brasil. Essa busca inicial mostrou que o termo “coronavírus” era mais frequentemente utilizado. Portanto, para este trabalho, o termo “coronavírus” foi associado a palavras-chave retiradas dos títulos das *fake news* para verificar se houve um crescimento no número de buscas ao longo do período de interesse. Na pesquisa, os termos foram utilizados na grafia correta e, fazendo uso do símbolo “+” como interseção, em grafias alternativas. O “+” funciona como um operador booleano, mais precisamente o “OR”, informando ao sistema de busca como combinar os termos de pesquisa. Isso faz com que o resultado apresente pelo menos uma das variações de escrita da palavra, ampliando o resultado da pesquisa (por exemplo: “uso de álcool em gel” + “uso de alquingel” + “uso de alcool gel” ou “coronavírus” + “coronavirus” + “corona vírus”).

O Google Trends permite, ainda, a classificação das buscas por sub-região, mostrando onde o termo pesquisado teve mais popularidade durante o período especificado. Para tanto, o número total de buscas por determinada palavra-chave em certa região geográfica é dividido pelo número total de buscas na região durante o período escolhido (23). Os resultados são apresentados de forma escalonada (0 a 100), onde o valor de 100 representa o pico de popularidade de um termo. Um valor de 50 significa que o termo teve metade da popularidade. Uma pontuação de zero significa que não havia dados suficientes sobre o termo, ou seja, não havia classificação das buscas realizadas na sub-região em relação ao total de buscas na grande região geográfica (23).

Utilizando as combinações de termos construídas com base nos títulos das notícias, foram identificadas as três principais datas com resultados de buscas acima de zero; essas três datas foram contrastadas às datas de divulgação das *fake news* estudadas, de forma a verificar se havia coincidência entre a publicação de *fake news* e o aumento do volume de buscas com termos vinculados. Finalmente, foram selecionadas para análise mais detalhada 10 notícias cuja publicação coincidiu com um aumento nas buscas de acordo com o Google Trends e que poderiam ter impacto negativo na saúde.

Por se tratar de um estudo de revisão documental, cuja característica é o manejo de dados secundários, e pelo fato de as buscas digitais terem sido realizadas em *sites* que veiculam informações disponíveis ao público (G1, Ministério da Saúde e Google Trends), o projeto não exigiu aprovação ética.

## RESULTADOS

Identificaram-se 339 *fake news* relacionadas à pandemia de COVID-19. Dessas, excluíram-se 10 duplicatas. Sendo assim, 329 *fake news* foram analisadas (253, ou 76,9%, do G1; e 76, ou 23,1%, do *site* do Ministério da Saúde).

Em relação às categorias temáticas, as *fake news* foram agrupadas da seguinte forma: economia (por exemplo, “rede de lojas Renner anunciou fechamento definitivo de lojas e demissão em massa de funcionários em meio a pandemia”), tratamento (por exemplo, “água com alho recém-fervida cura o coronavírus”), surgimento do vírus (por exemplo, “coronavírus foi criado por cientistas”), xenofobia e racismo (por exemplo, “chinesa com coronavírus é presa em mercado na Austrália após cuspir em bananas”), política (por exemplo, “WhatsApp limita encaminhamento de mensagens no Brasil após pressão política”), epidemiologia e estatística (por exemplo, “Hospital das Clínicas de São Paulo vazio em meio a pandemia”), auxílios (por exemplo, “doação de combustível para trabalhadores pela Petrobras em meio a pandemia”), crime (por exemplo, “fiscais da Prefeitura de São Paulo são agredidos por ambulantes em meio a pandemia de COVID-19”), penalidades e punições por descumprimento das normas sanitárias (por exemplo, “policiais agredindo cidadãos por descumprirem isolamento social”), sintomatologia (por exemplo, “coronavírus causa necessariamente inflamação na garganta”), predição do futuro (por exemplo, “edição de revista publicada em 2003 falava do novo coronavírus”), comportamento do vírus (por exemplo, “coronavírus não resiste ao calor e à temperatura de 26ºC”), posicionamento de pessoas famosas (por exemplo, “Tiago Leifert e equipe do BBB festejando em restaurante em meio a pandemia do coronavírus”), meio ambiente (por exemplo, “flamingos ocuparam canal de Veneza em meio à pandemia do coronavírus”) e prevenção (por exemplo, “chá de erva-doce e fígado de boi previnem contra o novo coronavírus”). A categoria prevenção deu origem a duas subcategorias, utilização de meios preventivos e contraindicação do uso de meios preventivos. A distribuição das *fake news* em cada categoria aparece na tabela 1.

**TABELA 1.** Análise das *fake news* sobre a pandemia de COVID-19 que circularam no Brasil de 1º de janeiro a 30 de junho de 2020

Variáveis	No.	%
Portal G1	253	76,9
*Site* Ministério da Saúde	76	23,1
Total	329	100,0
Categorias		
Política	66	20,1
Epidemiologia e estatística	64	19,5
Prevenção	53	16,1
Tratamento	39	11,9
Xenofobia e racismo	18	5,5
Auxílios	17	5,2
Economia	17	5,2
Penalidades e punições por descumprimento das normas sanitárias	10	3,0
Posicionamento de pessoas famosas	10	3,0
Predição do futuro	10	3,0
Surgimento do vírus	9	2,7
Crime	7	2,1
Sintomatologia	5	1,5
Comportamento do vírus	3	0,9
Meio ambiente	1	0,3
Canal de divulgação		
Mídias sociais/redes sociais (origem não identificada)	130	39,5
WhatsApp	100	30,4
Facebook	69	21,0
Twitter	13	4,0
*Site*	8	2,4
Instagram	6	1,8
YouTube	2	0,6
TikTok	1	0,3
Formato de divulgação		
Imagem	107	32,5
Mensagem (WhatsApp, Messenger etc.)	92	28,0
Vídeo	77	23,4
Texto (textos postados em redes sociais)	30	9,1
Sem informação	6	1,8
Link	6	1,8
Áudio	4	1,2
Multimídia	3	0,9
Carta	2	0,6
Tabela	1	0,3
Documentário	1	0,3

***Fonte***: G1 – Fato ou Fake – Coronavírus; e Ministério da Saúde – Fake News – Coronavírus.

**FIGURA 2.**
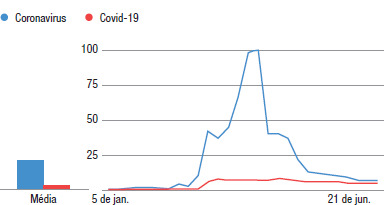
Buscas no Google Trends utilizando os termos “coronavírus” e “COVID-19”, de janeiro a junho de 2020, Brasil^a^

***Fonte***: Google Trends.

a Data de pico: 12 a 19 de abril de 2020.

Em relação aos meios de divulgação, 130 (39,5%) *fake news* foram divulgadas em mais de um canal ou rede social, não sendo possível identificar onde se iniciou a disseminação. Entre as *fake news* com fonte de disseminação identificável, 100 (30,4%) foram disseminadas por meio do WhatsApp e 69 (21%), via Facebook (tabela 1). Os formatos mais frequentes de divulgação das *fake news* foram imagens, mensagens de texto e vídeos (tabela 1).

Observou-se aumento de 34,3% nas buscas para 113 conjuntos de termos encontrados nas 329 *fake news*. Considerando as regiões do Brasil, houve aumento marcante nas buscas utilizando 60 (45,1%) termos no Sudeste, 37 termos (27,8%) no Nordeste, 18 termos (13,5%) no Sul, 12 termos (9%) no Centro-Oeste e seis termos (4,5%) no Norte. Na comparação entre os termos “coronavírus” e “COVID-19” no Google Trends, o termo “coronavírus” foi o mais utilizado pelos usuários (figura 2). Entre as 10 *fake news* selecionadas para uma análise mais detalhada, quatro eram sobre formas de tratamento, como a utilização de alimentos milagrosos (por exemplo, feijão da Igreja Mundial e ingestão de enxofre). A hidroxicloroquina e o chá de erva-doce apareceram mais de uma vez em épocas diferentes (tabela 2).

## DISCUSSÃO

As *fake news* são um tema altamente relevante no cenário político e social brasileiro. A expressão traz consigo uma contradição: a palavra *fake*, como adjetivo, altera a própria natureza do substantivo *news*, já que, em princípio, seria de esperar que as notícias veiculassem apenas informações verídicas (24, 25). No entanto, o conceito foi popularizado por governos que se mantêm em luta contra a imprensa (25). Entre as categorias de *fake news* mais frequentemente encontradas neste estudo, está a política, seguida pela categoria de epidemiologia e estatística, de tratamento e de prevenção da COVID-19.

Reflexões feitas por Linsey McGoey trazem à luz a investigação das formas multifacetadas pelas quais a ignorância estratégica pode ser aproveitada (26). McGoey define a ignorância estratégica como “a habilidade de explorar o desconhecimento para ganhar mais poder” (27, p. 1) e como a decisão de pessoas, empresas e governos de ignorar informações para benefício próprio (27). Nesse contexto, o fenômeno das *fake news* representa uma ferramenta acessível a governos e outros grupos para desviar, obscurecer, ocultar ou moldar o conhecimento de acordo com os seus interesses (26). Fantasiadas de jornalismo, as *fake news* contribuem para aumentar a descrença na ciência e nas instituições de saúde pública. Além disso, outros estudos evidenciaram a disseminação deliberada de notícias falsas sobre a COVID-19, como a promoção da hidroxicloroquina como um possível “tratamento precoce” e a ideia de isolamento vertical como argumento contra o distanciamento social (28).

**TABELA 2.** Principais *fake news* sobre COVID-19 que circularam no Brasil de 1º de janeiro a 30 de junho de 2020

Data de publicação no portal	Título	Data de aumento no Google Trends	Portal	Esclarecimento
29/jan/2020	Chá de erva-doce é tratamento e cura o novo coronavírus	-15/mar/2020 -25/abril/2020	Ministério da Saúde	Nenhum tipo de chá pode ser utilizado para substituir um tratamento adequado contra a gripe, muito menos contra a COVID-19. O chá de erva-doce não contém a mesma substância do medicamento Tamiflu. Além disso, o Hospital das Clínicas de São Paulo, citado no texto da mensagem, esclareceu que não realizou alertas à população.
30/jan/2020	Vitamina C e zinco funcionam como forma de prevenção contra o novo coronavírus	-26/jan/2020 -18/abril/2020	Ministério da Saúde	Até o momento da veiculação da notícia, não havia nenhum medicamento específico ou vacina que pudesse prevenir a infecção pelo novo coronavírus.
28/fev/2020	Álcool em gel não funciona como forma de prevenção contra o coronavírus	-23/fev/2020 -2/maio/2020	G1	A Anvisa reforça que a lavagem de mãos com água, sabão e álcool em gel 70% é o procedimento padrão mais recomendado na literatura médica para prevenção de infecção não somente pelo coronavírus, mas por outros agentes patogênicos.
23/mar/2020	Aplicativo Coronavírus-SUS, do governo do Brasil, é inseguro	-15/mar/2020 -16/maio/2020	Ministério da Saúde	O aplicativo Coronavírus-SUS-COVID-19 foi desenvolvido pelo Ministério da Saúde utilizando todos os padrões de segurança e preza pela confidencialidade das informações de seus usuários.
23/mar/2020	Governo do Brasil anuncia vacina do coronavírus	-15/mar/2020 -23/maio/2020	Ministério da Saúde	Muitas pesquisas estão sendo desenvolvidas para o combate à COVID-19, entretanto, até o momento da veiculação da notícia, não havia nenhum medicamento, substância, vitamina, alimento específico ou vacina que pudesse prevenir a infecção pelo coronavírus.
29/mar/2020	Feijão da Igreja Mundial cura o coronavírus	-15/mar/2020 -18/abril/2020	G1	O Ministério da Saúde informou que não havia, até o momento de veiculação da notícia, produto, substância ou alimento que garantisse a prevenção ou tratamento do coronavírus. Conforme determinação do Ministério Público Federal, o Ministério da Saúde esclareceu que é falsa a informação sobre cura ou prevenção da COVID-19 a partir do plantio de sementes de feijão comercializadas pelo líder da Igreja Mundial do Poder de Deus.
3/abril/2020	OMS fez cartaz recomendando “evitar sexo desprotegido com animais”	-15/mar/2020 -13/jun/2020	G1	A imagem, que tem circulado principalmente em inglês, foi manipulada digitalmente. A palavra *sex* (sexo) foi colocada no lugar de *contact* (contato). O cartaz verdadeiro está no *site* da Organização Mundial da Saúde e não fala de sexo com animais.
21/maio/2020	Pesquisa recente indica a hidroxicloroquina como o tratamento mais eficaz contra o coronavírus	-15/mar/2020 - 9/maio/2020	G1	A Organização Mundial da Saúde diz que a cloroquina pode causar efeitos colaterais e não tem eficácia comprovada no tratamento da COVID-19. Não há evidências científicas de que o medicamento funcione para esse fim.
9/jun/2020	Enxofre destrói o coronavírus	-24/maio/2020 -27/jun/2020	G1	O consumo de enxofre não é recomendado para esse fim e, ainda, dependendo da concentração, tem poder tóxico. Nenhum estudo até agora descreveu a eficiência de tratamentos baseados em compostos derivados de enxofre para a COVID-19.
30/jun/2020	Termômetro digital infravermelho causa câncer e cegueira	-22/maio/2020 -9/maio/2020	G1	O uso desse tipo de termômetro é seguro. Existem vários tipos de raios infravermelhos. O utilizado em termômetros, pelo comprimento da onda, baixa potência e baixo tempo de exposição, não leva a malefícios para a retina. Além disso, o infravermelho é usado em alguns tratamentos oncológicos. O aparelho tem sido usado para evitar a propagação da COVID-19 no comércio.

***Fonte***: *Site* G1 – Fato ou Fake – Coronavírus; e *site* do Ministério da Saúde – Fake News – Coronavírus.

De modo mais geral, observou-se que, no período de 2015 a 2019, os sentimentos de dúvida e desconfiança sobre a importância da vacinação foram impulsionados por informações de origem política disseminadas *on-line*, que colaboraram para o crescimento da pauta antivacina em vários países (29). Além disso, o conteúdo das *fake news* manipula valores individuais, no sentido de que as pessoas acreditam naquilo que convém aos seus interesses políticos, sociais e até religiosos, independentemente de escolaridade (26). Em situações de medo e incerteza, como na pandemia, as pessoas tendem a acreditar no que causa conforto, mesmo quando não há comprovação científica; um exemplo são as *fake news* sobre alimentos milagrosos para tratamento ou prevenção da COVID-19 (30). Fato semelhante foi observado em um estudo que analisou 1 225 *fake news* e constatou três tipos comuns de desinformação relacionados à COVID-19: alegações falsas, teorias da conspiração e terapias de saúde pseudocientíficas, sendo a última acerca de métodos de prevenção e tratamento para a COVID-19 (31).

As *fake news* disseminadas pelos meios digitais relacionadas à COVID-19 tem o potencial de influenciar o comportamento da população, prejudicando sua adesão aos cuidados comprovados pela ciência. Em um cenário pandêmico, os efeitos são ainda mais devastadores, uma vez que pesquisas apontam que 110 milhões de cidadãos brasileiros (mais de 50% da população do país) acreditam em notícias falsas sobre a COVID-19 (18). As *fake news* na categoria de epidemiologia e estatística se caracterizam por afirmações falsas sobre o número de casos e óbitos e sobre mortes por outras causas sendo computadas como sendo de COVID-19, bem como por afirmações que comparam a COVID-19 a uma “gripezinha” que dispensa o isolamento social (32). Essa categoria também engloba outras afirmações que permeiam o negacionismo e o que veio a ser chamado de necropolítica no país (33).

Em relação às macrorregiões, o Sudeste alcançou o maior número de buscas no Google Trends. Do mesmo modo, observou-se, nos resultados do Google Trends, que as regiões Sudeste (45,1%) e Nordeste (27,8%) apresentaram aumento repentino das buscas relacionadas a *fake news* até a 26ª semana epidemiológica. Segundo os dados obtidos por pesquisadores do Centro Regional de Estudos para o Desenvolvimento da Sociedade da Informação (CETIC), o Sudeste concentra o maior (73%) número de domicílios com acesso à Internet (34), enquanto o Nordeste detém o menor percentual (57%) de domicílios com acesso. Entretanto, por serem as regiões mais populosas, o Sudeste e Nordeste apresentam o maior número absoluto de domicílios com conexão à Internet (34). As buscas mais frequentes detectadas pelo Google Trends nessas regiões não podem ser explicadas pela escolaridade das pessoas. Vale notar que foram essas as regiões com os maiores índices de casos e óbitos no Brasil até a semana epidemiológica 26 (27 de junho de 2020), enquanto as regiões Centro-Oeste e Sul apresentaram os menores índices (1). Essa simultaneidade poderia sugerir alguma ligação entre a propagação das *fake news* e o número elevado de casos e óbitos em cada região.

Pesquisadores do CETIC evidenciaram que o uso do WhatsApp está em ascensão em todo o país, sendo que indivíduos na faixa etária de 25 a 34 anos representam 96% dos usuários, e indivíduos com 60 anos ou mais representam 86% (34). Nosso estudo identificou o WhatsApp como o meio mais frequente de divulgação de *fake news*, corroborando os dados obtidos pelo aplicativo Eu Fiscalizo (vinculado à Fundação Oswaldo Cruz [FIOCRUZ]), no qual verificou-se circulação de 73,7% dos conteúdos falsos no WhatsApp (19). Entre os danos causados pelas *fake news*, destacam-se a perda de confiança em instituições outrora reconhecidas e legitimadas socialmente como canais de apresentação de fatos verídicos, como a grande imprensa e a academia (9-11), o aumento de casos e óbitos pela difusão de práticas comprovadamente ineficazes (9-11) e o potencial incremento de custos nos sistemas de saúde.

Os resultados obtidos nas buscas do Google Trends em relação aos equipamentos de proteção e cuidados de saúde demonstram o potencial de alcance das notícias e a possibilidade de previsão do comportamento de tais indivíduos de acordo com suas buscas (22). O aumento das buscas com termos relacionados às *fake news* demonstra a relação entre a disseminação de um assunto e a utilização de determinados termos em buscas (9-11), além de fornecer indícios do impacto da *fake news* na população, potencialmente acarretando os problemas provenientes de tal prática, já discutidos anteriormente.

A identificação dos tipos mais comuns de informações enganosas e suas principais fontes durante uma pandemia permite reconhecer as esferas onde proliferam as *fake news* e tem o potencial de auxiliar as instituições de saúde pública na identificação de estratégias de combate à desinformação. Foi interessante notar, neste estudo, que o portal G1 — Fato ou Fake continua inserindo notícias que desmentem as *fake news* relacionadas ao coronavírus. Em contraste, o portal do Ministério da Saúde não atualiza o registro de *fake news* desde 8 de junho de 2020, não sendo localizada a aba de *fake news* no *site* do Ministério da Saúde (em 30 de setembro de 2020). Além disso, a notícia que negava a eficácia do uso da cloroquina no tratamento da COVID-19 foi apagada no mesmo dia em que o Ministério da Saúde autorizou o uso do medicamento (1).

Na interpretação dos resultados aqui apresentados, é importante salientar as limitações do estudo. Primeiro, embora agregue dados de buscas ao longo do tempo, o Google Trends evidencia apenas o comportamento de busca dos indivíduos que utilizam o mecanismo de pesquisa do Google. Dessa forma, este estudo não aborda o comportamento de busca em outras plataformas. Além disso, não há informação sobre os métodos usados pelo Google Trends para gerar os dados a partir dos algoritmos de busca. Observamos também que a pesquisa produzia resultados distintos pela simples modificação de um acento ou de uma palavra da norma culta para um termo de uso coloquial. A limitação de termos de pesquisas agrupados também se constituiu em dificuldade para a obtenção de dados. Ademais, é importante notar a utilização de apenas duas fontes de informação para identificação das *fake news* neste estudo, o que pode ter gerado resultados subestimados em relação ao número de *fake news* divulgadas durante o período pesquisado.

Finalmente, vale ressaltar que as autoridades públicas e os meios de comunicação oficiais são essenciais para o combate efetivo às *fake news* (9-11). Os profissionais de informação em saúde e os jornalistas devem tomar medidas para auxiliar o público a identificar o discurso por trás das *fake news*, além de evidenciar a necessidade de averiguar a informação recebida antes de compartilhá-la com terceiros (31), juntamente ao processo de divulgação científica por meio de *hashtags, podcasts* e outros formatos.

Em conclusão, as *fake news* divulgadas durante os primeiros 6 meses da pandemia de COVID-19 no Brasil se caracterizaram, principalmente, por conteúdos de posicionamento político e desinformação sobre número de casos e óbitos e medidas de prevenção e de tratamento. Os principais veículos de divulgação das *fake news* foram o WhatsApp e o Facebook, com utilização de mensagens, imagens e vídeos, tendo maior alcance nas regiões Sudeste e Nordeste do país.

## Declaração.

As opiniões expressas no manuscrito são de responsabilidade exclusiva dos autores e não refletem necessariamente a opinião ou política da RPSP/PAJPH ou da Organização Pan-Americana da Saúde (OPAS).
